# Exosomes as New Biomarkers and Drug Delivery Tools for the Prevention and Treatment of Various Diseases: Current Perspectives

**DOI:** 10.3390/ijms22157763

**Published:** 2021-07-21

**Authors:** Qi Liu, Shiying Li, Amandine Dupuy, Hoa le Mai, Nicolas Sailliet, Cédric Logé, J.-Michel H. Robert, Sophie Brouard

**Affiliations:** 1 Department of Pharmacology, College of Pharmacy, Dalian Medical University, Dalian 116044, China; liuqi.paris@free.fr (Q.L.); lsy199549@dmu.edu.cn (S.L.); 2 Unite Mixte de Recherche 1064, Centre de Recherche en Transplantation et Immunologie, Inserm, CHU Nantes, Université de Nantes, ITUN, F-44000 Nantes, France; amandine.dupuy1@etu.univ-nantes.fr (A.D.); Le.Hoa-Mai@univ-nantes.fr (H.l.M.); Nicolas.sailliet@etu.univ-nantes.fr (N.S.); 3 Institut de Recherche en Santé 2, 22, Cibles et Médicaments du Cancer et de l’Immunité IICiMed-AE1155, Nantes Atlantique Universités, Université de Nantes, Boulevard Bénoni-Goullin, F-44000 Nantes, France; Cedric.Loge@univ-nantes.fr

**Keywords:** exosomes, biomarkers, drug delivery tools, therapeutic target, diseases

## Abstract

Exosomes are nano-sized vesicles secreted by most cells that contain a variety of biological molecules, such as lipids, proteins and nucleic acids. They have been recognized as important mediators for long-distance cell-to-cell communication and are involved in a variety of biological processes. Exosomes have unique advantages, positioning them as highly effective drug delivery tools and providing a distinct means of delivering various therapeutic agents to target cells. In addition, as a new clinical diagnostic biomarker, exosomes play an important role in many aspects of human health and disease, including endocrinology, inflammation, cancer, and cardiovascular disease. In this review, we summarize the development of exosome-based drug delivery tools and the validation of novel biomarkers, and illustrate the role of exosomes as therapeutic targets in the prevention and treatment of various diseases.

## 1. Introduction

Extracellular vesicles (EVs) are lipid bilayer-bound particles secreted by most living cells [1]. Although these molecules have long been considered mediators of cellular waste, the discovery of their involvement in intercellular communication is generating increasing interest in many biological fields [2]. In addition, EVs allow the selective transport of functional proteins, nucleic acids (DNA, miRNA, mRNA), lipids or small molecules while protecting them from enzymatic degradation by the environment and facilitating their intercellular uptake [3,4]. These vesicles have been found in almost all biological fluids that allow their transport, such as plasma [5] or urine [6]. These EVs can then be taken up by neighboring or more distant cells in which they mediate many physiological or pathological processes by modulating their phenotype. EVs are classified into three types based on their biogenesis, size and surface markers: apoptotic bodies, microvesicles and exosomes. Due to their endosomal origin, exosomes are considered to play a key role in biological processes in normal and pathological conditions. Because of their inherent characteristics, such as stability, biocompatibility and stealth ability, exosomes are considered an interesting target for disease treatment. Exosomes are involved in basic physiological processes such as neuronal communication [7], antigen presentation [8], immune responses [9], organ development [10] and reproductive performance [11] by transmitting microRNAs, proteins, long-chain noncoding RNAs, circular RNA and DNA to mediate signal transduction between adjacent or distal cells. These structures are also involved in pathological diseases, such as cancer progression [12], cardiovascular disease and [13], inflammation [14] and even facilitate viral infection [15] and prion transmission [16].

Exosomes have become a new drug delivery tool due to their many advantages compared with traditional delivery systems. Efficient loading of external drugs or molecules into exosomes is another demanding and challenging task [17]. Like synthetic nanoparticles, several methods, including direct mixing, incubation, sonication, vortexing, remote loading, electroporation, and transfection, can be applied to load micro- and macromolecules into exosomes. For some hydrophobic drugs (e.g., curcumin), EVs can be loaded with the drugs by direct mixing [18]. Paclitaxel can be loaded by mixing and sonication [19,20]. Due to the presence of the lipid bilayer around the exosome perimeter, electroporation is widely applied to load nucleic acids (siRNAs) [21]. It has been reported that describe a vesicular stomatitis virus G (VSVG) pseudo typing-based approach to load EV membranes with the receptor-binding domain (RBD) of the viral spike protein, which can be used to deliver antiviral drugs against SARS-CoV-2 infection [22].

The study of the expression and regulation of exosomes can contribute to improvements in understanding, diagnosis, and therapy of diseases. This paper reviewed the literature on the occurrence, action, mechanism and potential therapeutic effects of exosomes in diseases. In addition, the current knowledge of the therapeutic applications of exosomes is summarized.

## 2. Exosomes Biology

### 2.1. Formation, Structure and Circulation

EVs are classified into three types based on their biogenesis, size and surface markers: apoptotic bodies, microvesicles and exosomes [23]. Apoptotic bodies are the largest category of vesicles. They are 50 to 5000 nm in size and are released during cell fragmentation in the late phase of apoptosis. Therefore, they contain mainly double-stranded DNA [24]. Microvesicles are intermediate-sized particles of 50 to 1000 nm that originate directly from the plasma membrane. Microvesicle arises through outward budding and fission of plasma membrane and is the result of dynamic interplay between phospholipid redistribution and cytoskeletal protein contraction. Membrane budding/vesicle formation is induced by translocation of phosphatidylserine to the outer-membrane leaflet through the activity of aminophospholipid translocases [25]. They are generated following the activation or delivery of a stimulus, leading to an influx of calcium and remodeling of membrane phospholipids by the enzymes scramblase and floppase. This mechanism leads to the external budding and membrane scission of these vesicles, which are then released into the extracellular environment. Due to their biogenesis mechanism, these vesicles are enriched in phosphatidylserine and membrane proteins [26].

**Figure 1:**
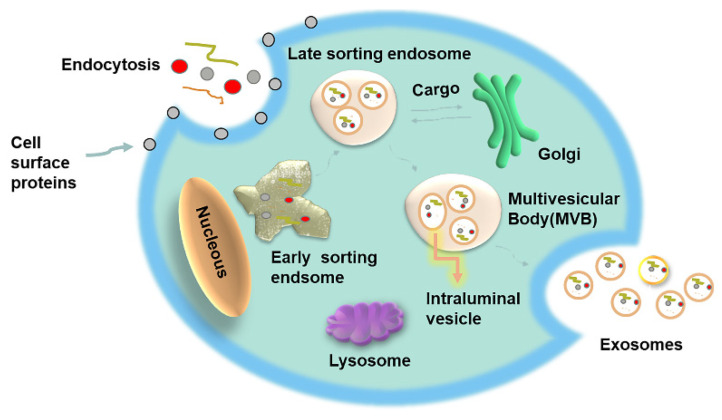
Exosomes biogenesis. Exosomes are formed by inward budding from the endosomal membrane, which leads to the formation of multivesicular bodies (MVBs). MVBs can be fated for lysosomal degradation or fusion with the plasma membrane, which is associated with the release of exosomes.

First found in sheep reticulocytes in 1983, exosomes were named by Johnstone in 1989. Exosomes can be differentiated from microvesicles and apoptotic bodies by their origin and scale. They are biological nanoscale lipid bilayer vesicles secreted by cells that originate from polyvesicles [27] that are released from the cell after fusion with the plasma membrane. These vesicles are approximately 40–100 nm in diameter with a density of 1.13–1.19 g mL^−1^ [28,29,30,31,32]. Exosomes circulate freely and can accumulate in different body fluids and function as carriers of a variety of functional biomolecules. Therefore, exosomes may become a promising noninvasive liquid biomarker and play an important role in clinical diagnosis and treatment in the future [33,34]. The formation of exosomes is a continuous process that mainly includes endocytosis, fusion and efflux. Initially, endosomal membrane invagination forms multivesicular bodies (MVBs) with dynamic subcellular structures. Multivesicular bodies have two outcomes: (1) fusion with lysosomes, followed by degradation of the lumen-like vesicles in them and (2) fusion with the cell membrane, followed by degradation of the lumen-like vesicles in the polycysts [35]. The initial endosome is formed by the invagination of the early cell membrane, and bioactive substances begin to accumulate in early sorted endosomes (ESEs). Then, under the control of the endocytosis sorting complex and other related proteins needed for transport, early endocytosis results in the late sorting endosome (LSE). After the second indentation, LSEs finally form polyfoam (MVBs), which contain many intraluminal vesicles (ILVs) [36]. After the fusion of MVBs and the cell membrane, intracellular substances are released to the outside of the cell in the form of vesicles [37]. After being released, exosomes may exist around the releasing cell or remain in the extracellular space. Additionally, they can move dynamically in body fluids. Exosomes can also be absorbed by adjacent and distant cells, thus changing the behaviors of target cells [38] (Figure 1).

### 2.2. Exosomes: Biogenesis and Cellular Absorption

The formation of ILVs within MVBs is enabled by three mechanisms: a mechanism dependent on the recruitment of endosomal sorting complexes required for transport (ESCRT) [39], a mechanism dependent on tetraspanin enrichment [40], and a ceramide-dependent mechanism [41]. Although in the ESCRT-dependent pathway, sorting of loaded proteins into ILVs can be performed through their ubiquitination [42], tetraspanins are also involved in the regulation of exosomes loading [43]. However, these mechanisms remain poorly understood. Due to their biogenesis pathway, exosomes show enrichment of proteins from the endosomal endocytosis pathway, such as TSG101 and ALIX, and of CD9, CD81 and CD63, but their enrichment in nucleic acids has also been highlighted, suggesting an active sorting mechanism of these molecules [44]. The transport of ILVs is enabled by the joint action of Rab27 a and b, GTPases, allowing the transport of ILVs along microtubules and facilitating their docking to the plasma membrane [45]. ILVs will then be released as exosomes into the extracellular environment through fusion of the plasma membrane and MVBs, probably mediated by the SNARE complex following rearrangement of the actin cytoskeleton [46]. Exosomes can act by different mechanisms on their target cell: either by direct binding between a molecule present on the surface of EVs and a receptor on the plasma membrane or by internalization. There are six types of internalization: macropinocytosis, phagocytosis, clathrin-dependent endocytosis, caveolae-dependent endocytosis, lipid raft-mediated endocytosis, and more rarely by direct membrane fusion. However, the fate of exosomes contents in the accepting cell remains unclear. These EVs will either release their material into the intracellular environment or be internalized into early endosomes. In the latter case, the early endosomes will mature and form EVs. These EVs will then either be recycled and resecreted or degraded after fusion of the MVE with a lysosome to support the metabolism of the recipient cell [47] (Figure 1).

### 2.3. Exosomes Classification

Since MISEV2014 [48], the growing recognition of the existence of many different types of EVs, of different sizes and cellular origins, has led to the publication of several studies. Some studies compared EVs recovered by medium speed centrifugation [49,50]. Another study used differential filtration to separate large microvesicles retained by 0.65 micron filters, and small “exosomes” passing through 0.1 micron filters [51]. Others further separated the high speed pellet to identify subpopulations of small EVs bearing different surface markers such as A33 antigen (*GPA33*) vs. EPCAM [52], lipid moieties binding Cholera Toxin, Annexin-V or Shiga Toxin [52], or tetraspanins CD63, CD9, and/or CD81 [53].

**Figure 2:**
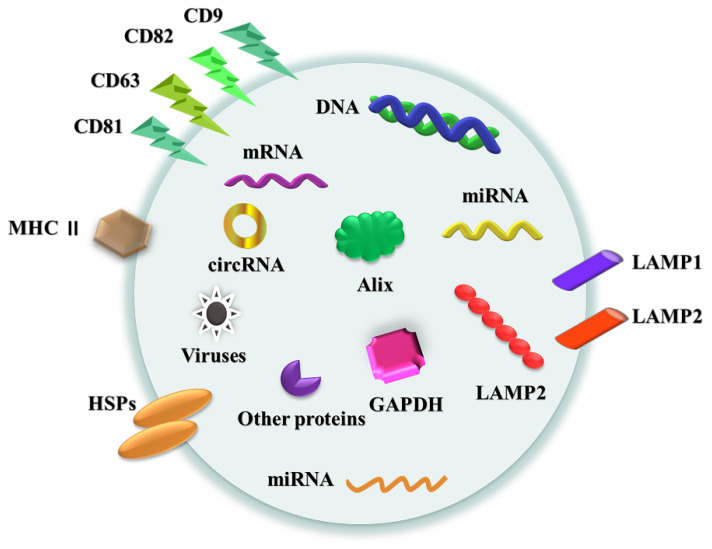
Exosomes contain proteins, nucleic acids, and other substances. Their proteins include heat shock proteins (HSP70, HSP90), four-transmembrane protein superfamily proteins (CD9, CD63, CD81, CD82), ESCRT complex-related proteins (Tsg101, Alix), glyceraldehyde-3-phosphate dehydrogenase (GAPDH), lysosomal associated membrane protein (LAMP1, LAMP2) and other proteins. Their nucleic acids include mRNA, miRNA, circRNA, DNA and so on. The other substances include specific components, which are closely related to their progenitor cells, that is, cell-specific molecules, such as MHC-II derived from antigen-presenting cells.

At present, exosomes are mainly classified on the basis of source. This classification does not take into account the characteristics and functional applications of various types of exosomes. According to high-throughput exosomes studies, exosomes contain many molecules, including proteins, lipids, metabolites, mRNA [54], mitochondrial DNA [55], miRNA [54] and many other noncoding RNAs [56] (Figure 2). Exosomes can be found in blood plasma/serum [57], saliva [58], breast milk [59], cerebrospinal fluid [60], urine, and semen [61]. According to whether exosomes have been artificially modified, they are divided into natural exosomes and engineered exosomes. Because the exocrine system functions under normal and tumor conditions, the exocrine system of animal origin can be divided into normal exocrine and tumor exocrine systems. Almost all types of normal cells, such as human umbilical vein endothelial cells, mesenchymal stem cells (MSCs), T cells, B cells, macrophages, dendritic cells (DCs), and natural killer cells (NK), can produce exocrine bodies [62,63,64,65]. Tumor cells can secrete many tumor exocrine bodies, and the specific antigens on their surface can reflect the properties of donor cells. Therefore, tumor exosomes have attracted much attention in cancer research. Tumor exosomes not only play an important role in tumor growth, metastasis and immunomodulation [66,67] but also can be used to monitor the development of the disease [68]. In addition, further subdivisions can be considered in terms of organophilia, biological distribution and immunogenicity in the future [37].

## 3. Involvement of Exosomes in Disease Immunopathology

### 3.1. Exosomes and Tumor Environment

In recent years, the role of exocrine circRNA in regulating tumor cell proliferation in various kinds of cancers has been identified. In colorectal cancer, circIFT80 promotes the development of colorectal cancer by entering exosomes, promotes DNA synthesis and inhibits apoptosis through the miRNA-1236-3p/HOXB7 axis [69]. The expression of circFMN2 in serum exosomes of patients with colorectal cancer is high and negatively correlated with the level of miRNA-1182. The combination of circFMN2 and miRNA-1182 can significantly promote the proliferation of colorectal cancer cells, which suggests that exocrine circFMN2 plays an important role in promoting the tumor growth of colorectal cancer [70].

Understanding the immune-suppressive or immune-activating role of exosomes present in the tumor microenvironment can ultimately lead to the identification of exosome-based biomarkers of response and to the design of rational combinatorial therapies [71]. Programed death ligand 1 (PD-L1), also known as differentiation cluster 274 (CD274) or B7 homologue B7 homologue 1, is a type I transmembrane protein encoded by the CD274 gene, which is formed by immunoglobulin V-like and C-like extracellular domains [72]. PD-L1 is widely expressed in various cell types, mainly in tumor cells, monocytes, macrophages, natural killer (NK) cells, dendritic cells (DCs) and activated T cells. This molecule can also be expressed in immune privileged areas (such as the brain and cornea) and retinas [73]. Recently, cancer-derived exosomes were shown to transfer functional PD-L1 and inhibit immune responses [69]. Further, in melanoma patients receiving PD-1 blockade, exosomal PD-L1 levels correlated with tumor burden and response to therapy. It is unclear whether exosomal PD-L1 directly correlates with tumor or immune PD-L1 status, but it may have utility as a predictive biomarker for PD-1 blockade. PD-L1-containing exosomes may be both regulators and biomarkers of therapy resistance. In short, exosomal PD-L1 has a vital function in tumor metastasis, immune escape, and immunotherapy, but it is not clear whether the function of exosomal PD-L1 is cancer type-dependent. Further clarification of the role of exosomal PD-L1 in tumor progression will contribute to the early diagnosis and treatment of cancer (Table 1).

Exosomes can mediate molecular communication and substance transfer between primary tumor sites and distant metastatic sites. Exocrine bodies play an important role in tumor cell metastasis and invasion by regulating a series of cellular activities, including epithelial-mesenchymal transformation (epithelial-mesenchymal transition, EMT) [9]. The results of studies on circRNA and gastric cancer show that circNRIP1 can be transmitted between gastric cancer cells through exocrine bodies. In addition, miRNA-149-5p sponges components of the Akt1/mTOR signaling pathways, thus promoting gastric cancer cell metastasis [76]. Some exocrine circRNAs play an important role in the progression and metastasis of pancreatic cancer. Li et al. [77] found that exocrine circPED8A is highly expressed in pancreatic cancer and is related to lymphatic invasion, TNM stage and low survival rate. CircPDE8A can promote the growth of tumor cells by upregulating the expression of MET (one of the key oncogenes of epithelial tumors). In addition, circPDE8A secreted by tumor cells can be released into the blood circulation through exosomes to regulate MACC1 as a miRNA-338 sponge and promote invasive metastasis through MET/mitogen-activated protein kinase 1 (mitogen activated protein kinase-1-MAPK1) or the protein kinase B pathway. In addition, scholars [78] have found that exocrine circIARS secreted by pancreatic cancer cells is widely expressed in pancreatic cancer tissues, and its expression level is positively correlated with liver metastasis, vascular invasion and TNM stage (liver metastasis: paired 0.011; vascular invasion: paired 0.020; trans TNM: paired 0.023). CircIARS can enter human microvascular endothelial cells through exosomes derived from pancreatic cancer cells, downregulate the levels of miRNA-122 and tight junction protein-1 (zonula occludens-1), upregulate the levels of RhoA and RhoA-GTP, increase the expression of F-actin and adhesion plaques, increase endothelial monolayer permeability and promote tumor invasion and metastasis. In addition, related studies on colon cancer and cholangiocarcinoma have identified a role of exocrine circRNA in promoting tumor invasion and metastasis.

### 3.2. Exosomes and Digestive Environment

As one of the important functional vectors of gastric cancer (GC), exosomal RNA plays an important role in the initiation and development of gastric cancer by promoting cell-to-cell communication between gastric cancer cells and the microenvironment [79]. Relevant studies have shown that exosomes are an important part of the tumor microenvironment in gastrointestinal cancer tissue and can promote the proliferation and metastasis of cancer cells, stimulate tumor angiogenesis, and inhibit the immune response of the host [80]. In addition, exosomes can effectively improve the accuracy and targeting of drug therapy for gastrointestinal cancer [81]. In conclusion, exosomes, especially exosome-derived miRNAs, play an important role in regulating the biological behavior of gastrointestinal cancer and have many advantages, such as good stability and convenient detection. *Helicobacter pylori* (Hp) infection is the most important factor leading to GC. Recent studies have shown that exosomes are associated with the occurrence of Hp-related diseases, having a tumor-promoting effect on tumor-associated macrophages, and promote GC progression [82]. Other studies have shown that exosomes in the conditioned medium of human gastric epithelial cells are involved in Hp infection [83]. This finding also shows that miRNA-155 exosomes from HP-infected macrophages can immunomodulate the inflammatory response and inhibit gastritis. Thus, exosomes play a key role in the diagnosis and treatment of gastrointestinal cancer.

### 3.3. Exosomes and Cardiovascular Diseases

Exocrine bodies are closely related to the occurrence and development of cardiovascular diseases such as hypertension, atherosclerosis, pulmonary hypertension, myocardial infarction, and myocardial hypertrophy. The cardiovascular system is an important site for intercellular transmission of exosomes. MicroRNA levels of exosomes related to cardiovascular disease, including miR-499, miR-133, miR-208, miR-192, miR-194, and miRNA-34a, are upregulated in patients with acute myocardial infarction and heart failure. Exosomes [84,85,86] can act on adjacent or remote target cells and mediate intercellular signal transduction. In addition, in pulmonary hypertension, researchers found that exosomes can ease pulmonary remodeling and reduce pulmonary hypertension by inhibiting high value-added pathways such as transcription factor-3 and inhibiting inflammation of monocytes [87].

### 3.4. Exosomes and Glioblastoma

Glioblastoma (GBM), also known as grade IV astrocytoma, is the most aggressive primary intracranial tumor of the adult brain [88]. Glioblastoma tumor cells release exosomes containing mRNA, miRNA and angiogenic proteins [12]. miRNAs have been found to function as regulatory molecules, acting as oncogenes or tumor suppressors and play prognostic roles in malignant transformation (including in GBM) and have been identified as novel therapeutic targets [89]. Previously, it had been shown that miR-125b overexpression decreased expression of cell cycle regulatory proteins such as CDK6 and CDC25A in U251 glioma cells, thereby preventing cell cycle arrest at the G1/S transition [90]. Another study showed that miR-181a, miR-181b and miR-181c act as tumor suppressors in GBM and contribute to the complexity of the pathological progression of glioma [91,92]. As with other cancers, miRNAs have great promise as prognostic biomarkers and therapeutic targets in GBM. miRNAs can function as potential oncogenes or tumor suppressors in gliomas [88].

### 3.5. Exosomes, the Endocrine System and Cancer

Recent studies have shown that exosomes secreted by cytotoxic T (TC) cells contribute to tumor progression, angiogenesis and metastasis. Exosomes in liquid biopsies can reflect the overall molecular information of the tumor and have natural advantages in the diagnosis of TC [93]. The advantage of miRNAs in diagnosis is that they are highly stable, protected by bilayer membranes, and contain key information related to the tumor biological response [37,93]. Lee et al. found that the levels of miR-146b and miR-222 in epithelioid cell (TPC-1) exosomes were higher than those in Nthy-ori3-1 (NTHY) cells, indicating that these two miRNAs may be biomarkers of follicular papillary thyroid carcinoma (PTC) recurrence [94]. Interestingly, another study detected plasma exosomes in PTC patients with or without lymph node metastasis, confirming that circulating exocrine miR-146b-5p and miR-322-3p have high diagnostic value in predicting lymph node melanoma metastasis (LNM) in patients with PTC [95]. Samsonov et al. compared patients with benign thyroid nodules and found that miR-31 expression was significantly upregulated in serum exocrine tissues of patients with PTC. [96] In addition, similar changes were found in miR-21 in the serum exocrine system of patients with follicular thyroid cancer (FTC). In addition, compared with that of FTC patients, the level of miR-21 in serum exosomes of PTC patients was lower, but the content of miR-181a-5p was significantly increased. Therefore, miRNAs in these exosomes can be used as diagnostic markers for PTC and FTC [97]. With the continuous improvement of high-throughput detection technology, more miRNAs have been found. Wang ZY et al. carried out plasma miRNA spectrum analysis in patients with PTC and healthy subjects and verified the experimental results. Among the candidate miRNAs, miR-346, miR-34a-5p and miR-10a-5p levels were upregulated in PTC plasma exosomes [98]. Pan Q isolated exocrine bodies from the plasma of patients with PTC and nodular goiter by small RNA sequencing and comprehensive analysis and identified a group of plasma exocrine miRNAs as candidate biomarkers for the diagnosis of thyroid nodules, among which miR-5189-3p was the best in the diagnosis of PTC. Dai D et al. found that miR-485-3p and miR4433a-5p may be used as biomarkers for the diagnosis of PTC. Plasma exocrine miR-485-3p can distinguish between high-risk and low-risk PTCs [99]. By analyzing exocrine bodies from different patients and screening a group of miRNAs in plasma exocrine bodies, Li MH et al. found that the combination of these miRNAs was more effective than any single marker in identifying PTC and thyroid nodules [100]. These results suggest that the comprehensive detection of various exocrine contents may be more advantageous.

### 3.6. Exosomes and the Urinary System

It has been reported that exocrine lncRNA-p21 inhibits the occurrence of prostate cancer and the expression of p53 transcriptional regulatory genes [101]. When binding to the DNA binding domain of glucocorticoid receptors, lncRNA-GAS5 inhibits antiapoptotic genes, thereby preventing prostate cancer [102]. However, renal EVs can also mediate several other pathological conditions, such as renal fibrosis and inflammation [103,104].

### 3.7. Exosomes in Metabolic Diseases

Metabolic syndrome (MetS), obesity and diabetes mellitus, are clinically classified as metabolic disorders [105]. Recently, extracellular vesicles (EVs) have been emerging as a novel way of cell-to-cell communication that transfers fundamental information between the cells through the transport of proteins and nucleic acids. EVs, released in the extracellular space, circulate via the various body fluids and modulate the cellular responses following their interaction with the near and far target cells. Clinical and experimental data support their role as biomarkers and bio-effectors in several diseases including metabolic syndrome [106]. New evidence shows that exosomes with flotillin immunomodulatory functions may be involved in the occurrence and development of autoimmune diabetes. For one thing, islet-derived exosomes can activate the immune system and cause an autoimmune response [107]. For another, exocrine bodies originating from the immune system may lead to dysfunction and beta cell death [108]. Another study showed that exosomes released by human urine-derived stem cells can prevent podocyte apoptosis and promote cell survival and angiogenesis in rats with T1DM [109]. In addition to T1DM, exosomes also play a role in other autoimmune diseases, including rheumatoid arthritis, systemic lupus erythematosus and Sjogren’s syndrome [110]. One result showed that exosomes from adipose stem cells (ADSCs) improved insulin sensitivity and hepatic steatosis, and reduced obesity, when injected into obese mice [111]. Furthermore, AT macrophages (ATM) exosomes from obese mice have been shown to induce systemic insulin resistance and glucose intolerance in lean mice, and these factors are ameliorated in obese mice when ATM exosomes from lean mice are treated in obese mice [112]. MiR-155 is a repressor of the adipogenic transcription factor peroxisome proliferator-activated receptor γ (PPARγ) and has been suggested to be a key mediator of the effect of ATM exosomes on insulin resistance [112]. Taken together, these studies highlight the potential importance of exosome-mediated crossover between key metabolic tissues in regulating metabolism under physiological and pathophysiological conditions [113]. MiR-197, miR-23a, and miR-509-5p have now been identified as potential contributors to dyslipidemia in metabolic syndrome. In addition, a reasonable association between miR-27a and miR-320a and patients with metabolic syndrome and type 2 diabetes has also been found [114]. Therefore, EVs could be new biomarkers predictive of metabolic pathologies and new exploitable structures in therapy [115] (Table 2).

### 3.8. Exosomes in Viral Pathogenesis

Viruses use exocrine pathways to gain entry, spread, perform viral packaging, and escape from the host immune system [126]; because of the similarity of exocrine biogenetic pathways (ESCRT-dependent and independent), their fate (endocytosis, endocytosis and receptor-mediated uptake by target cells) and viral uptake, packaging and release are comparable to those of relatives [127]. Viral infection stimulates host cells to secrete exocrine bodies, which act as pathogen-related molecular models, carry inflammatory mediators, and cause inflammation [128]. HCV mRNA in exosomes induces secretion of interferon alpha (IFN alpha) from macrophages, and exosomes from C3/36 cells infected with Zika virus induce expression of tumor necrosis factor alpha (TNF alpha) from monocytes and cause endothelial damage to induce intravascular coagulation and inflammation. Exosomes from Kaposi sarcoma-associated herpesvirus also cause endothelial damage and induce the expression of IL6 [129]. Exosomes from virus-infected cells also cause apoptosis of immune cells. The 2019 coronavirus disease (COVID-19) caused by severe acute respiratory syndrome coronavirus 2 (SARS-CoV-2) was first reported in December 2019. It is believed that COVID-19 may be transmitted from person to person through droplets, fecal transmission and direct contact with aerosols. A relatively high basic fecundity (R 0) value estimated between 2.2 and 5.7 caused the virus to spread rapidly, resulting in a pandemic [130]. COVID-19 is a highly contagious respiratory syndrome that can cause multiple organ failure and may lead to death in a small number of infections. The virus can replicate in a variety of cells expressing ACE2, including nasal epithelium, nasopharynx, upper respiratory tract, type II lung cells in the lung, gastrointestinal tract, immune cells and endothelial cells [131,132]. Recent data have shown that lipid metabolism, including cholesterol metabolism [133], is involved in the pathogenesis of COVID-19, raising the question of whether exosomes are involved in the pathogenesis of SARS-CoV-2 infection. Consistent with this idea, SARS-CoV-2 protein interaction group analysis revealed interaction with Rab protein, which is part of the ESCRT pathway involved in exocrine biogenesis. In short, exosomes from virus-infected cells can cause tissue damage by activating inflammation and cytotoxicity. For example, HIV infection induces secretion of exosomes that are enriched in viral Nef protein [134]. Likewise, Epstein–Barr virus (EBV)-infected cells secrete exosomes enriched with galectin 9 that cause apoptosis of cytotoxic T cells specific to EBV-infected cells [135] (Table 3).

### 3.9. Exosomes in Transplantation

Transplantation is the treatment of choice for many terminal organ failures. However, it comes with an important risk of chronic rejection. Exosomes are key mediators of donor recognition by the host immune system through protein transfer of the preformed donor MHC-peptide complex in host APC that subsequently activates donor-specific T cells [140]. Moreover, studies focusing on blocking this phenomenon are increasing and show promise. However, exosomes derived from host immune cells have shown interesting capacities to modulate rejection, as in other pathological conditions. Exosome-based therapies are currently being studied to specifically silence the immune system toward the graft. Several cell types are candidates for sources of exosomes: mesenchymal stem cells, regulatory T cells, M2 macrophages and immature dendritic cells, which are well-known immunoregulatory cells [141,142,143,144].

### 3.10. Anti-Inflammatory and Antimicrobial Vesicles

Mesenchymal stem cells (MSCs) can interact with the immune system to prevent infection through both direct and indirect mechanisms [145]. MSCs, exosomes secreted by these cells can be used as complementary antimicrobial agents, as a substitute for or in combination with antibiotics under specific physiological conditions or specific priming conditions [146]. In particular, antimicrobial properties are associated with the paracrine of several antimicrobial peptides (AMP), which have a wide range of antimicrobial properties, as well as specific extracellular vesicle (EV) secretion, including the release of immunomodulatory factors MSCs that retain antimicrobial properties [147] and are considered safer than parental cell administration [148]. EVS as a cell-free agent and/or drug carrier may have therapeutic effects for sepsis [148] and may be developed as a superior drug delivery vehicle [149].

EV number, size and their biologically active material is altered in numerous inflammatory conditions and EV can alter the cellular functions of neutrophils, monocytes, macrophages and their precursor hematopoietic stem and progenitor cells (HSCs) [150]. Neutrophils can release at least two sub-classes of EV, termed: neutrophil derived trails (NDTRS), which are generated by integrin mediated interactions by migrating neutrophils in response to vascular wall forces and neutrophil derived microvesicles (NDMV), which are dependent on the PI3K pathway and released by membrane blebbing following neutrophil activation [151,152]. Mesenchymal stem cell (MSC) EV modulate neuroprotection during ischemic injury by inhibiting neutrophil recruitment and mediate similar protective effects to those observed with neutrophil depletion [153]. Monocyte-derived EV may provide utility as diagnostic biomarkers for the assessment of pathologies where monocyte phenotypes contribute to the inflammatory disease such as infection, dyslipidemia, diabetes, obesity and cardiovascular diseases [154]. In conclusion, EV, especially exosomes, can be used as the carrier of K, which is expected to improve the therapeutic effect and reduce adverse reactions [155].

## 4. Exosomes as a Means of Drug Delivery

In addition to their utilization as therapeutics, exosomes have shown different capacities as biomarkers of rejection. Exosomes content has been observed in heart, lungs, kidney, liver, and bone marrow transplanted patients within proteomic and transcriptomic studies, and multiple biomarkers of chronic rejection have been identified, mostly associated with tissue damage and immune activation [156].

### 4.1. Exosomes and Chemical Synthesis

Pascucci et al. loaded exosomes derived from MSCs with paclitaxel by culturing MSCs with a high dose of paclitaxel [157]. These paclitaxel-loaded exosomes effectively inhibited the proliferation of pancreatic cancer cells. It was confirmed that exosomes derived from MSCs can package and transport substances. Furthermore, it has also been proven that exocrine bodies from different cell sources have specific markers, which have the potential for disease diagnosis. Jang et al. used U937 monocytes as donor cells and cocultured them with 400 μg/mL doxorubicin [158]. U937 cell-derived exosomes loaded with doxorubicin were obtained by ultracentrifugation. Although anticancer drug-loaded exosomes have inhibitory effects on cancer cells, their safety and efficiency in drug delivery need to be further studied. The natural molecular structure of exosomes provides the basis for the uptake and transport of small molecular chemical drugs, reduces the toxicity and side effects of drugs, improves the utilization efficiency of drugs, and has natural advantages over traditional chemical drug delivery systems. Moreover, exosomes can be engineered for better delivery of their loading either through the expression of organotropic proteins that direct exosomes toward the tissue of interest [159] or by reshaping or resizing the exosomes to access specific sites, such as the tumor microenvironment. Exosome modifications can also affect their half-life by hiding them from the immune system through pegylation [160] or by decreasing their clearance using scaffold proteins [161].

### 4.2. Exosomes and Peptidomes

#### 4.2.1. Protein

According to proteomic studies, exosomes not only have specific proteins that depend on the type of secreted cells but also have a specific subset of cellular proteins found in exosomes, regardless of cell type [162]. The protein content of exocrine proteins has been well identified by a variety of proteomic techniques. High-throughput proteomic analysis revealed proteins related to cell structure, exercise and adhesion, such as actin, myosin, radionuclide, tubulin, integrin and cell surface receptors, including epidermal growth factor receptor (EGFR), platelet-derived growth factor receptor β (PDGFRB) protein and plasminogen activator urokinase receptor (PLAURs), and signal transduction proteins. Transcription factors and metabolic enzymes were identified [163,164]. These proteins are involved in some basic cellular processes, such as cell adhesion, structural dynamics, membrane fusion, metabolism and signal transduction [165]. In addition, four transmembrane proteins and integrins (such as CD63, CD9, CD81 and CD82) are essential for cell targeting and adhesion, while Rab GTPases, annexins and flotillin are involved in membrane transport and fusion [30]. CD9 [166] is primarily known as a specific exosomes marker. CD9 expression is sometimes correlated with better survival and is sometimes used as a biomarker of invasion and late stages. CD9 is reported to suppress motility and to promote adherence, leading to the suppression of tumor progression. Indeed, CD9 expression is often downregulated in advanced stages of cancer [167]. ICAM1/CD54 and the cell surface peptidases CD26 and CD13 were also present in exosomes. In addition, these structures also contains cytokines, transcription factor receptors, growth factor receptors and other bioactive molecules [168]. Exosomal protein composition can vary between different cell types and under different culture conditions, there are two main mechanisms of protein sorting: dependent and independent endosomal transport complex (ESCRT). ESCRT consists of four polymer complexes, namely, ESCRT-0 to ESCRT-III. In recent years, studies have shown that exosomes as drug carriers can carry a variety of proteins to achieve targeted drug delivery and stabilize enzyme activity. Mizrak et al. [169] first reported that proteins were loaded into exocrine bodies for antitumor therapy. This study successfully loaded a mixture of protein and mRNA into exosomes, which led to the study of exocrine-carrying proteins. Subsequently, a study of exocrine catalases also confirmed that exocrine bodies can carry exogenous catalase, which can be used in the treatment of Parkinson’s disease (Parkinson’s disease, PD) [170]. In addition, Sterzen-Bach et al. [171] constructed an exocrine delivery system carrying recombine and achieved brain-targeted drug delivery across the blood–brain barrier. This study used a molecular switching mechanism to realize the loading of exogenous Cre recombinant enzymes in exocrine systems, which provides a new idea for the construction of exocrine delivery systems carrying exogenous proteins. Yuan et al. [172] used exosomes secreted by natural macrophages to carry brain-derived neurotrophic factor (BDNF) to achieve active and targeted drug delivery under brain inflammation, which provides a new method for brain inflammation treatment and brain nutrition. Although initial research to achieve targeted treatment of central nervous system diseases is common, research on nonbrain targeted drug delivery has also increased in recent years. For example, Malhotra et al. [173] constructed an exocrine delivery system carrying transferrin and whey ferritin and achieved tumor-targeted drug delivery for systemic drug delivery.

#### 4.2.2. Nucleic Acids

Valadi and his colleagues reported for the first time that mast cell-derived exosomes contain mRNA and miRNA [54]. In addition, there are other noncoding RNAs that can transfer between cells and may regulate gene expression in receptor cells. Exosomes contain a specific subset of cellular RNA, which is not related to the cell origin. Exocrine bodies produced by endothelial cells promote angiogenesis in a small RNA-dependent manner. These findings suggest that a particular RNA is actively rather than passively classified as foreign. Researchers found that mRNA with a 3’-UTR can be packaged into exocrine regions by specific RNA fragments [174]. Dicer and Ago2 are key components of miRNA processing, and they have been found to be functionally present in the exocrine system. There is also a tetranucleotide sequence in miRNA, which controls their location in exosomes. Similarly, there is a tetranucleotide sequence that can induce miRNAs to be packaged into exosomes and recognized by heterogeneous ribonucleoproteins [175]. Recently, it has been shown that another similar RNA-binding protein, SYNCRIP, is the cytoplasmic RNA-interacting protein of synaptic binding molecules, which controls the sorting of mRNA into exosomes [176]. The protein binds to a specific miRNA library by recognizing a specific miRNA motif and loading it into exosomes, which may provide a potential way for selective regulation of exocrine cargo.

#### 4.2.3. Lipids

Lipids are essential components of exosomal membranes, and it is well-known that specific lipids are enriched in exosomes compared to their parent cells [177]. The capacity of exosomal lipids and their derivatives to interact with recipient cells makes these molecules important mediators of disease progression [178]. For example, it was found that exosomes isolated from bronchoalveolar lavage fluid of asthmatic patients contained significantly lower phosphatidylglycerol (PG), ceramides and ceramide phosphates, resulting in altered airway surfactant compositions, impaired immune signaling, and consequently, reduced lung function [179]. Furthermore, the myoblast cells C2C12 exposed to palmitate produced palmitate-enriched exosomes with the capability to induce myoblast proliferation and to alter the expression of genes involved in cell cycle and muscle differentiation [180]. A different mechanism for exosomal lipid-mediated regulation has been proposed in the human pancreatic tumoral SOJ6 cell line, opening new perspectives for exosome-based cancer treatment [181]. Exosomal lipids as therapeutic targets have proposed several strategies to highlight their potential as new therapeutic targets by inhibiting key aspects of their biology, such as biogenesis, release and cellular uptake [182]. One of the strategies proposed with this aim is the reduction of the endosomal sorting and exosomes biogenesis through the inhibition of the sphingomyelinase. This inhibition can be achieved with the blood-pressure-lowering drug amiloride, which has demonstrated an efficient in vivo reduction of the circulating tumor-derived EVs with the subsequent reduction in tumor growth [41].

### 4.3. Exosomes and Natural Medicine Monomers

Natural medicine monomers (NMMs) are effective components in natural drugs, which have single structure and inherent biological activity in a variety of disease models [183]. Plant exosomes are rich in miRNAs, studies have shown that exosomes can maintain the stability of miRNAs in the decoction process of traditional Chinese medicine [184] and deliver miRNAs and other substances to target cells to mediate a variety of physiological and pathological processes [28]. An increasing number of reports have shown that miRNAs not only perform biological functions in their original system but also regulate transboundary gene expression [185]. In some studies, the natural medicine active monomer miRNA was confirmed to be transported across borders [186]. In addition, we often eat plant foods, and its miRNAs can also be detected and stable in human blood [187] and have a specific pharmacological effect. However, there are few studies on plant exosomes related to natural medicine active monomers. At present, the pharmacological mechanism of exocrine bodies of some traditional Chinese medicine plants and their small RNAs is not clear, so it is extremely important to study the exocrine bodies of natural medicine active monomers and their small RNAs.

## 5. Conclusions

In recent years, extracellular bodies have attracted much attention due to their ability to target drug delivery. It has been shown that exosomes are promising as a potential tool for clinical diagnosis and treatment. As a non-invasive diagnostic component in medical practice, exosomes can provide a useful biomarker library for a variety of diseases as an important part of circulating biomarkers [188]. Although exocrine therapy has made some breakthroughs, such as inhibiting exocrine biogenesis and secretion may help to reduce the occurrence of tumors. However, there are some limitations and challenges in translating it into clinical treatment. Therefore, further research will help to target exocrine biogenesis and prevent tumorigenesis.

In addition, tumor-derived exosomes (TDEs) play a key role in the establishment and progression of tumors and are emerging biomarkers for tumor diagnosis in personalized medicine [189]. Studies have shown that the recognition of unique binding peptides of exosomes released by multiple myeloma may be a very sensitive diagnostic method for clinical assessment of disease progression [190]. However, there is currently a lack of effective exosomes isolation and characterization technology platform. However, effective techniques for isolating and characterizing exosomes are currently lacking. Therefore, it is necessary to find a universally definable exosomes marker to reveal the diversity of exosomes.

To date, exosome-based nanocarriers have been developed for the treatment of many prevalent and stubborn diseases, delivering small molecule drugs and bioactive macromolecules. Therefore, in order to accelerate the routine use of exosomes as carriers for effective and safe drug delivery to target cells, there is an urgent need for new and improved techniques to effectively load therapeutic drugs into exosomes [191].

In addition, it has been reported that plant liquids and active monomers of natural drugs also play a unique role in the treatment of diseases, but the pharmacological mechanism of active monomers and small molecule RNA of some natural drugs is still not clear [192], so the study of active monomers of natural drugs is also very important. Finally, although exocrine therapy can inhibit tumor growth, the goal of complete tumor eradication has not been achieved. Therefore, the exact cause and mechanism remain to be discussed.

## Figures and Tables

**Table 1:** Function of exosomal PD-L1 in tumor progression.

Type of Tumor	Source	Function	References
Colorectal cancer (CRC)	Serum and plasma	MiR-486-5p promotes the proliferation and migration of CRC cells by activating the signal pathways of pleomorphic adenomatoid gene 2 (PLAGL2), insulin-like growth factor 2 (IGF2) and β-catenin in vivo and in vitro.	[74]
Head and neck squamous cell carcinomas	Plasma	Downregulate CD69 expression on effector T cells to inhibit antitumor response	[75]
Prostate cancer	Tumor tissue	Suppress the function of T cells in the draining lymph node and block anti-PD-L1 antibodies	[66]
Melanoma	Plasma	Suppress the function of CD8 + T cells and cause failure of anti-PD-1 therapy	[69]

**Table 2:** The targets of exosomes in diseases.

Disease	Exosomal miRNAs	Target or Pathway	References
Acute myeloid leukemia	Exosomes with MICA/B (MHC I chain-related proteins A and B)	By downregulating NKG2D receptor expression	[116]
Brain cancer	Brain endothelial cells	Rhodamine 123, PTX, DOX	[117]
Breast cancer	MiR-365 in macrophage-derived exosomes	The triphospho-nucleotide pool, the enzyme cytidine deaminase	[118]
Leukemia	MiR-210	CD107a	[119]
Lung cancer	MiR-494	Suppresses PTEN (PTEN (phosphatase and tensin homolog deleted on chromosome ten), it is located at 10q23.3 and the transcriptional product is 515 kb mRNA).	[120]
Colorectal cancer	MiR-31-5p in (tumor-derived exosomes) TDEs	LATS2	[121]
Nasopharyngeal cancer	MiR-24-3p	ND	[122]
Esophageal cancer	MiR-21 in TDEs	PDCD4	[123]
Head and neck cancer	MiR-196a in cancer associate fibroblasts (CAF)- derived exosomes	CDKN1B and ING5	[124]
Pancreatic cancer	MiR-106b in CAFs-derived exosomes	TP53INP1	[125]

**Table 3:** Exosomes in the pathogenesis of viral infections.

Virus	Source	Function	References
Avian influenza (H5N1)	miR-483-3P	Increased production of proinflammatory cytokines in vascular endothelial cells	[136]
HIV	Nef	Susceptibility to infection and apoptosis of CD4 cells	[134,137]
KSHV	miRNA and others	IL6 production and cellular metabolism	[129]
Coronavirus	CD9	Proviral	[138]
EV-A71	Viral protein and nucleic acid	Virus spread	[139]

## Data Availability

Not applicable.
